# What impact does written information about fatigue have on patients with autoimmune rheumatic diseases? Findings from a qualitative study

**DOI:** 10.1002/msc.1164

**Published:** 2016-11-18

**Authors:** Ruth I. Hart, Wan‐Fai Ng, Julia L. Newton, Katie L. Hackett, Richard P. Lee, Ben Thompson

**Affiliations:** ^1^ University of York York UK; ^2^ Newcastle University Newcastle‐upon‐Tyne UK; ^3^ Newcastle‐upon‐Tyne Hospitals NHS Foundation Trust Newcastle‐upon‐Tyne UK

**Keywords:** fatigue, rheumatic diseases, written information, qualitative research

## Abstract

**Objectives:**

Although fatigue is a common symptom for people with rheumatic diseases, limited support is available. This study explored the impact of written information about fatigue, focusing on a booklet, *Fatigue and arthritis*.

**Methods:**

Thirteen patients with rheumatic disease and fatigue were recruited purposively from a rheumatology outpatient service. They were interviewed before and after receiving the fatigue booklet. Two patients, plus six professionals with relevant interests, participated in a focus group. Transcripts were analysed thematically and a descriptive summary was produced.

**Results:**

Interviewees consistently reported that fatigue made life more challenging, and none had previously received any support to manage it. Reflecting on the booklet, most said that it had made a difference to how they thought about fatigue, and that this had been valuable. Around half also said that it had affected, or would affect, how they managed fatigue. No one reported any impact on fatigue itself. Comments from interviewees and focus group members alike suggested that the research process may have contributed to the changes in thought and behaviour reported. Its key contributions appear to have been: clarifying the booklet's relevance; prompting reflection on current management; and introducing accountability.

**Conclusions:**

This study indicated that written information can make a difference to how people think about fatigue and may also prompt behaviour change. However, context appeared to be important: it seems likely that the research process played a part and that the impact of the booklet may have been less if read in isolation. Aspects of the research appearing to facilitate impact could be integrated into routine care, providing a pragmatic (relatively low‐cost) response to an unmet need.

## 1 INTRODUCTION

As previously documented in *Musculoskeletal Care (*Farren, Goodacre, & Stigant, 2012 *)* and elsewhere (Hewlett, Cockshott, Byron, Kitchen, & Tipler, 2005; Mengshoel, Norheim, & Omdal, 2014; Overman, Kool, Da Silva, & Geenen, 2016; Schoofs, 2001), fatigue is a significant and burdensome symptom for people with autoimmune rheumatic diseases. It appears to reduce health‐related quality of life substantially and may in some instances have a greater impact than the more widely attended to symptom of pain (Kirwan & Hewlett, 2007). However, despite the prevalence and impact of fatigue, effective care strategies are yet to be established and clinicians often struggle to address it when raised (Repping‐Wutts, van Riel, & van Achterberg, 2008). Although there is evidence to suggest a biological as well as a psychosocial basis for fatigue (Newton & Jones, 2010), pharmacological therapies appear to have limited effect, even where they have proved effective for pain and inflammation (Chauffier, Salliot, Berenbaum, & Sellam, 2012; Ng & Bowman, 2010).

Non‐pharmacological strategies have been found to benefit some patients with fatigue and other long‐term conditions (Patterson, Wan, & Sidani, 2013). A systematic review (Cramp et al., 2013) found some evidence of benefit for psychosocial interventions and physical activity in managing fatigue linked to rheumatoid arthritis (RA). Group programmes, delivered by clinical psychologists and underpinned by cognitive behavioural therapy (CBT), have been judged as showing particular promise (Hewlett et al., 2011); research is under way to explore whether other rheumatology professionals might deliver such programmes to similar effect (Hewlett et al., 2015). However, although promising, such programmes are unlikely ever to be available, accessible and attractive to all patients in need of education and support (Thompson, 2011).

Other patients may turn, or be directed, to self‐management resources in the shape of written information in either print or electronic form. These materials have featured as the ‘usual care’ arm of trials of group programmes (Hewlett et al., 2011, 2015). However, despite appearing to offer a pragmatic solution to the information and support needs of patients unable or unwilling to access group programmes, there is little evidence that such materials are a widespread and consistent feature of usual care.

The use of patient information materials in trials (Hewlett et al., 2011, 2015) is providing useful outcome data in the form of clinical and other measures. However, information on the processes involved – how patients perceive, interact with and ultimately employ such resources – remains limited. With the exception of the early work of Bishop, Barlow, Williams, and Hartley (1997), patient literature (in rheumatology) has had surprisingly limited scrutiny in its own right. The present study set out to fill these potentially important knowledge gaps, by exploring patients' response to the Arthritis Research UK publication *Fatigue and arthritis* (Arthritis Research UK, 2011). This is a 24‐page booklet describing the features and causes of fatigue, and recommending a range of strategies to reduce its impact. The advice contained is broadly consistent with that provided online by the other organizations producing information for people with autoimmune rheumatic diseases (e.g. the US‐focused Arthritis Foundation). However, the booklet offers greater detail, is available as a hard copy and includes practical tools such as a chart for monitoring activity and fatigue.

## 2 METHODS

This paper reports the findings of a study, conducted in England over the period 2014–2015, investigating the reception, use and impact of the *Fatigue and arthritis* booklet by and on patients using a rheumatology outpatient service. The methodological approach taken was qualitative description, as described by Sandelowski (2000, 2010). This is a pragmatic, naturalistic approach to qualitative research, which focuses on producing low‐inference descriptions of experiences and events. It is particularly suited to producing ‘minimally theorized’ findings (Sandelowski, 2000) of practical value to practitioners and policymakers. As such, it fitted well with our ambitions for the project.

### 2.1 Sampling and recruitment

Qualitative description favours the construction of a non‐random sample reflecting the diversity of a given population (a goal often referred to as achieving ‘maximum variation’). Samples need to be of an adequate size to support this. Based on prior experience of treating and researching this patient group (Hart et al., 2015; Lee, Thompson, Whybrow, & Rapley, 2016; Thompson, 2011), the team predicted that a sample of 12–15 patients would be sufficient to accommodate potentially significant areas of variation and to achieve ‘data saturation’ (where no new themes, ideas or issues emerge). Ultimately, 13 patients were recruited for interview over a period of approximately 12 months. Two patients, one of whom had taken part in interviews, were recruited to the ‘expert’ focus group convened at the end of the study.

The sample was constructed purposively, with ongoing attention to diagnosis, gender, age and fatigue severity (see Table 1), as well as wider health, including mental health; social, occupational and domestic backgrounds; and life demands. Our concern was to ensure variety within the sample, so while fatigue severity was assessed using the Fatigue Impact Scale (FIS) (Fisk et al., 1994), no particular level of fatigue was set, a priori, as a condition for in−/exclusion. Instead, potential participants were eligible if they had been diagnosed with one of the inflammatory rheumatic diseases specified (ankylosing spondylitis, primary Sjögren's syndrome or rheumatoid arthritis), reported fatigue which they felt was significant, and their fatigue was judged to be related to the rheumatic disease and not to another condition (e.g. anaemia, hypothyroidism). Similarly, participants were asked to complete the Hospital Anxiety and Depression Scale (HADS) (Zigmond & Snaith, 1983) to enable us to assess variation in, and characterize more fully, the wider health of our sample.

**Table 1:** Characteristics of interviewees (*n* = 13)

Interviewee	Diagnosis^a^	Gender	Age (years)	FIS score^b^	HADS scores^c^ (A, D)
**A**	pSS	Female	77	63	4,4
**B**	pSS	Female	70	56	6,5
**C**	AS	Female	52	71	5,9
**D**	RA	Male	25	45	6,5
**E**	AS	Male	32	88	13,7
**F**	RA	Female	29	33	6,4
**G**	RA	Female	40	36	8,8
**H**	AS	Male	58	74	7,7
**I**	pSS	Female	70	61	5,9
**J**	pSS	Female	63	56	5,3
**K**	RA	Female	65	97	9,10
**L**	pSS	Male	61	99	11,8
**M**	AS	Female	57	78	8,3

a One of three specified inflammatory rheumatic diseases: primary Sjögren's syndrome (pSS); ankylosing spondylitis (AS); and rheumatoid arthritis (RA).

b The Fatigue Impact Scale (FIS) is a 40‐item questionnaire exploring three dimensions of fatigue. Scores may range from 0 to 160, with higher scores indicating higher fatigue impact.

c The Hospital Anxiety and Depression Scale (HADS) is a 14‐item questionnaire with two subscales [Anxiety (A) and Depression (D)] each running from 0 to 21. Scores of 0–7 are within the ‘normal’ range. Scores of 11 and above indicate a probable mood disorder.

Potential research participants were identified by clinical members of the research team during routine clinical encounters on the basis of their professional knowledge and with reference to patient records. As sampling progressed, and patients with more specific characteristics were sought, help was sought from other clinicians working in the rheumatology outpatient service. Initial approaches to prospective participants were made by clinicians, with expressions of interest subsequently followed up by the project researcher (the first author).

Six health professionals (with interests in musculoskeletal care, fatigue and/or patient education) were recruited to the study as focus group participants. The approach was again purposive and the group included two clinicians (from within and outwith rheumatology), a nurse and three allied health professionals (from occupational therapy, psychology and physiotherapy services). Professional participants were identified by the research team but approached directly by the project researcher. All potential participants (patients and professionals) were given written information on the study and in turn provided written consent.

### 2.2 Data collection

Data were collected through serial semi‐structured interviews (26) and a focus group (one). These methods support exploration of ‘complex phenomena’ (Tong, Sainsbury, & Craig, 2007) with interviews, in particular serial interviews, generating rich, contextualized data on individual experiences, beliefs and values (Lee et al., 2016; Murray et al., 2009; Ong & Richardson, 2006; Paskins & Hassell, 2012). Focus groups give access to different forms of expression, and perspectives arising as a consequence of social interaction (Kitzinger, 1995). The use of more than one method, or ‘methodological triangulation’, enriches understanding and supports validation (Denzin, 1989).

Patients were interviewed by the first author (a social scientist with prior experience of qualitative research in rheumatology) before and after being given a copy of the *Fatigue and arthritis* booklet. Interviews took place in settings chosen by interviewees (their homes, the hospital, university and a café). Initial (“pre‐booklet”) interviews explored patients' circumstances and their experiences of, and efforts to manage, fatigue. Follow‐up (“post‐booklet”) interviews typically took place around four to six weeks later and explored patients' impressions and use of the fatigue booklet, and the impact they perceived it having. Interview guides were drafted at the start of the study by the project researcher, with input from the wider team and patient partner. They were revised as the study progressed to take account of preliminary analyses and in response to the statements of each interviewee. It is more appropriate to view them as guides for conversation than as prescriptive scripts. The interviews, which lasted from 23 to 132 minutes, were all recorded and transcribed verbatim.

The focus group was convened in the final phase of the project in order firstly to explore whether interaction between patients and professionals might draw out alternative perspectives on the booklet (contrasting with each other or with those emerging in interview). Its secondary function was to invite critical reflection on the findings from the interviews and their potential implications for practice. All participants were sent a copy of the booklet ahead of the event and asked to read it by way of preparation. Topics explored include participants' views on the booklet, perspectives on the key findings from the interviews, and thoughts as to the future development and use of fatigue‐related educational resources. The focus group was facilitated by the first author and took place in a hospital meeting room. It ran for 90 minutes and was recorded and transcribed.

### 2.3 Data analysis

Data analysis ran alongside and informed data collection. Transcripts were checked for accuracy and then systematically analysed. The initial analysis, undertaken by the first author, involved line‐by‐line coding (Charmaz, 2006) to identify and abstract salient features of individual transcripts. Working within broad a priori themes (which also informed data collection and reporting), data and codes were then compared, sorted, related and (in the case of some codes) combined, until patterns, exceptions and revealing illustrations could be identified. Case summaries, charts, diagrams and memos were employed both to facilitate the process of analysis and to provide the wider team with access to the data and the analytical logic. Meetings with the wider team, which included a patient partner, and an external expert, encouraged reflexivity and improved analytical rigour.

### 2.4 Ethical approval

The study had Research Ethics Committee approval from the Proportionate Review Sub‐committee, National Research Ethics Service Committee Yorkshire & Humber‐Leeds East (ref. 14/YH/1054). It complied fully with the Declaration of Helsinki.

## 3 RESULTS

We begin this section by outlining how interviewees were affected by and tried to manage fatigue, before receiving the booklet. We then detail their responses to the booklet, and the changes in thought and behaviour they reported. We close by considering how the research experience itself may have contributed to those impacts. Data from the expert focus group are incorporated where they illuminate a point or provide an alternative perspective.

### 3.1 Experience and management of fatigue prior to receipt of the booklet

Our interviewees had varying histories of fatigue and rheumatic disease. For some, these were longstanding problems; for others, they were more recent developments. Although describing different patterns of fatigue, interviewees consistently reported that it made life more challenging and less rewarding. Fatigue disrupted activities and increased their physical and/or mental demands. Motivation to engage in social or leisure activities was undermined:

Socializing, just doing things that you want to do, are rather harder, or get put on hold, because you're tired all the time. [Interviewee D]

Often, people did not understand their fatigue, or connect it with their condition:

It hadn't occurred to me that it might be part and parcel of the condition. [Interviewee A]

Instead they attributed it to age, apathy or other – undiagnosed – illnesses. This lack of understanding left them feeling guilty and anxious about their work, domestic and social lives:

You feel like you're lazy, you know. I sort of come in and I'm thinking, you know, “Eeh, I'm such a lazy so‐and‐so”. [Interviewee C]

People worried about how their difficulties were perceived and judged by others, and the additional challenges the future might bring.

Interviewees described having made changes to their lives, to try to deal with fatigue. These included: conserving energy; managing demands by planning ahead; taking breaks for rest and recovery; and looking after themselves better. Some of these changes were active and pre‐emptive choices but others – such as rest – were often reactive – that is, precipitated by overwhelming fatigue. What emerged strongly from the data was that, even where people had identified helpful strategies, they struggled to use these consistently:

Some days, I handle it really badly… I won't pace myself, some days I, I still, just approach things badly, or just won't talk to people’. [Interviewee E]

No one reported having professional support to identify or implement fatigue management strategies.

Overall, fatigue‐related communication with health professionals (in primary or specialist care) appeared limited; for some, the initial interview was the first time they had talked about fatigue at any length. In addition to general difficulties with regard to communication in medical consultations, the data suggest a number of barriers specific to fatigue. These include: reliance on a colloquial vocabulary *(“*so tired”*, “*exhausted*”, “*knackered”*, “*wiped out”*, “*done in”); uncertainty about fatigue's relationship to rheumatic disease; doubt as to fatigue's ‘place’ on the consultation agenda; and a belief that nothing can be done. These barriers affected if and how concerns were shared, and could be reinforced by clinicians' reactions to disclosures of fatigue. Patients wanted professionals to initiate regular discussions:

It would be great if the consultants did say to you “And how are your fatigue levels?” But that doesn't happen. It doesn't happen. [Focus group participant (FGP) T]

### 3.2 Response to the booklet: Reported changes in thought and behaviour

When asked what impact the booklet had upon them, interviewees typically reported that it had made a difference to how they thought about fatigue, and that this was of real value. Understanding fatigue and its association with rheumatic disease helped to allay fears that fatigue was a sign of another, undiagnosed health problem or an inevitable age‐related decline:

The relief… of recognizing that it's part of the condition, not that I was sinking into an age‐related depression. [Interviewee A]

It validated interviewees' experiences and concerns, and somewhat alleviated the guilt associated with decreased activity:

‘Makes you feel a bit more like you're not making it up. [Interviewee F]

The booklet gave interviewees access to new ways of defining and describing their experience, enabling and encouraging the discussion of fatigue and its impacts:

I think I just need to be more honest, I suppose, and not try to cover up [Interviewee M]

Critically, it conveyed the message that it was possible to target important drivers of fatigue and, by doing so, reduce its impact:

There's things out there you can … incorporate into your life to make you feel better. [Interviewee G]

This sense that things could be done was a starting point, and a powerful motivator for change.

Interviewees largely valued the prompt to reflect on their current practice (i.e. efforts to maintain routines despite fatigue, or to manage fatigue):

It is useful now and again just to … think about it and maybe analyse it, analyse what you're doing, and if there is any, any changes that you can effect, because you tend just to go on with the same thing. [Interviewee H]

Around half said that the booklet had affected, or would affect, their approach to managing fatigue, and reported making, or planning to make, small but potentially significant adjustments to their behaviour. This included adopting practices broadly in line with ‘the four Ps’ (pacing, prioritizing, planning and problem‐solving (Arthritis Research UK, 2011)). Behaviours aligned with ‘pacing’ and ‘prioritizing’ featured in a particularly wide range of accounts, with interviewees describing new patterns of rest and activity, novel strategies for conserving energy and efforts to review and more vigorously prioritize commitments:

Just looking at what I do in a day … just trying to decide, really, “Yes, that needs to be done. That can wait. And that, it doesn't really matter if I don't do (it)”. [Interviewee G]

Several interviewees intended to monitor their energy output more closely and some had begun to schedule pleasurable as well as utilitarian activities:

We're just trying to like, go out and do things, trying to get out more [Interviewee L]

Other reported changes were efforts to improve general wellbeing through making more time for sleep, taking more exercise and attending more closely to diet.

While these interviewees were clear on the need for, and likely benefits of, change, many also identified barriers and challenges. We do not know how successfully these were circumvented, and whether all the intended changes were ultimately made. Although challenges were seldom framed as insurmountable, the need for support was emphasized. Challenges to initiating and maintaining the recommended behaviours were diverse, relating to: other symptoms and/or conditions; personal responsibilities and resources; individual psychology; and the clarity and immediacy of the “return” on the changes. One interviewee warned:

If it doesn't work in the first week, and make an, an instant difference, it's difficult to, to just really stick with it. [Interviewee E]

We noted that nobody reported any change in fatigue or its impacts by the time of the second interview. By contrast, interviewees often stressed that fatigue continued to impact negatively on their quality of life. Professionals attending the focus group said that this was to be expected, and that patients should be warned that in the short‐term their sense of fatigue might even increase:

One of the real blocks to people gradually doing more is the belief that hurt equals harm… They think, “Oh, my symptoms have got worse, I should stop”… You (need to) warn people that they'll get worse (before they get better). [FGP U]

### 3.3 Contribution of the research experience

Research participants (interviewees and focus groups members alike) saw the research project as providing a distinctive context for exposure to the booklet. In several instances, interviewees cited this as significant. The data more generally suggest three ways that the research process may have contributed to the changes in thought and behaviour reported. Firstly, it established the relevance of the booklet (with participant information documents explicitly linking fatigue and arthritis, and recruitment conversations reinforcing this). This was of obvious importance where interviewees had not previously connected fatigue with their condition. It was also helpful to those who had not named their experience “fatigue” or whose diagnosis did not feature in the booklet title:

What you're always looking for is something specifically about you… (And) it doesn't say ankylosing spondylitis anywhere on there. [Interviewee E]

Secondly, the line of questioning adopted in the “pre‐booklet” interview prompted patients to reflect on their current approaches to fatigue management. Interviewees were asked to describe, in some detail, their own strategies for managing fatigue and how effective these had been. The use of “How”, “What if” and “Why (not)” questions introduced gentle challenge. For several interviewees, this led to an acknowledgement that their current approach to managing fatigue was sub‐optimal, a logically necessary precursor to contemplating change. The third significant feature was commitment to follow‐up, in the form of the “post‐booklet” interview. Being held to account was cited as important by several interviewees:

If you hadn't been coming back would, would I have actually sat down and read the book from cover to cover, and actually, you know, give it the concentration I did? I probably wouldn't, I prob‐, I probably wouldn't, to be honest. [Interviewee H]

Focus group members also saw accountability as key:

With any information‐giving, it needs to be reviewed. [FGP Z]

Focus group members considered the potential for these research features to be reproduced in routine practice. The group agreed on the importance of rheumatology professionals drawing attention to the association between fatigue and rheumatic disease, and the potential to manage it using non‐pharmacological strategies. They suggested that the *Fatigue and arthritis* booklet could be introduced effectively in the context of such a conversation. They viewed it as both desirable and feasible to introduce an element of accountability (and advocated adding a template to the booklet for recording intentions or goals in support of this). They suggested that professionals could, and should, commit to discussing the booklet and patients' goals at future appointments; patients might be encouraged to identify a friend or family member who could hold them to account in the interim*.*

## 4 DISCUSSION

Recent years have seen information provision play an increasingly prominent role in health policy (Department of Health, 2012; Department of Health and Human Services (US), Office of Disease Prevention and Health Promotion, 2016; Washington & Lipstein, 2011). It has been conceptualized both as an intervention in itself and a central plank in shared decision‐making initiatives (Elwyn et al., 2010) and self‐management programmes (The Health Foundation, 2015). High‐quality information has been described as that which is relevant, evidence‐based, developed with users and embedded within care (Patient Information Forum, 2013). Increasingly, the case for investment in health information draws on “discourses” (Dixon‐Woods, 2001) of both patient education *and* patient empowerment. It has been argued to improve quality of care, service use and costs, patient outcomes and patient satisfaction (Department of Health and Human Services (US), Office of Disease Prevention and Health Promotion, 2016; Patient Information Forum, 2013).

*Written* information has been characterized as low‐cost, flexible and an effective aid to understanding and recall (Ellis, Hopkin, Leitch, & Crofton, 1979; Harris, Smith, & Veale, 2005; Patient Information Forum, 2013). However, some authors have warned that care should be taken not to overstate its effects and cautioned that different patient groups may not benefit equally (Blickem et al., 2011; Thompson, 2011). Ongoing disparities in access to written information in electronic form remain a concern to policymakers (Department of Health and Human Services (US), Office of Disease Prevention and Health Promotion, 2016). Furthermore, it has been questioned whether information alone can be relied upon to bring about behaviour change, and argued that theoretically grounded behavioural programmes have better outcomes (John et al., 2011). A recent publication by The Health Foundation (2015) reached the conclusion that information may increase knowledge, but that to influence behaviour other forms of support may be needed.

At first sight, our own research, which finds written information to have an impact both on thoughts and behaviours, appears at odds with this wider evidence base. However, taking into account the context in which the booklet was distributed and how, in consequence, people engaged with it, our findings become easier to reconcile. We explain this in more detail below. Then, having specified the conditions under which the booklet brought about change, we conclude by making some suggestions as to how its impact might be reproduced.

While it is clear that people need to encounter the booklet, they may either find it or be given it. Our data suggest that there are advantages to the latter, and that when people are given a booklet by a professional (in the case of our project, a researcher), they engage with it more actively. This resonates with claims made elsewhere (Thompson, 2011; Patient Information Forum, 2013) regarding the value of what the latter organization terms “infomediaries”. We have already noted how some patients with conditions other than (rheumatoid) arthritis expressed uncertainty, initially, as to whether the booklet was intended for them. In addition to confirming its relevance, professionals can frame engagement with the booklet in a number of important ways. In the present study, this included inviting patients, in the initial interview, to reflect on their prior experiences and management practices. In many instances, this led to recognition that their management practices were sub‐optimal and might be modified. It seems likely that this may be a pre‐condition for behaviour change. The Health Foundation (2015) have reported that the impact of written materials (and, indeed, of other forms of information and support) on self management, is maximized when backed up by professionals using techniques such as motivational interviewing (Elwyn et al., 2014; Treasure, 2004) to help patients to develop goals and solve problems in the course of consultations. Although the research interviews were not intended to take the form of motivational interviews, a key feature of that type of counselling – the expression of empathy through reflective listening (Treasure, 2004) – was a characteristic. In particular, the second interview provided a forum for people to reflect on their practices and how the advice contained in the booklet fitted with, had or might affect these. The serial nature of the interviews was, perhaps, the most significant feature of the research, with all participants expecting to be asked to give an account of their reaction to, and use of, the booklet. The role of follow‐up in supporting behaviour change is acknowledged in the literature (Sohl, Birdee, & Elam, in press). Active and sustained follow‐up of patients' self‐management goals (in addition to their clinical status) is also a key component of the “productive interactions” (Cramm & Nieboer, 2014) associated with Wagner's chronic care model (Wagner, 1998).

These features of the research are all potentially replicable in routine practice, and at relatively little cost. This is an important pragmatic consideration in the UK, where growth in demand for health services has not been matched by increases in resources (Roberts, Marshall, & Charlesworth, 2012). Health professionals, however, may themselves need support to make the most effective use of information materials such as the fatigue booklet (Department of Health and Human Services (US), Office of Disease Prevention and Health Promotion, 2016). Almost 20 years ago, after a wider review of educational materials for patients with arthritis, Bishop et al. (1997) stressed the importance of educating professionals in the use of patient literature, and guidelines on how to make the most effective use of leaflets were produced. Our findings suggest that there might be value in updating those guidelines and actively encouraging health professionals to use literature such as the *Fatigue and arthritis* booklet to support and enhance their patients' care.

### 4.1 Limitations of the study

We do not deny that our research has its limitations – most obviously, study duration and sample (size and character). Data regarding the impact of the booklet on patients' thoughts and/or behaviours do not suggest any difference by diagnosis. More nuanced differences in patient characteristics (e.g. education) and circumstances (e.g. life demands) may be significant, but our sample does not allow us to reach firm conclusions on this. There remains scope to characterize further the exact population to whom the benefits of the booklet – with and without additional support – might extend. Another important question is the extent to which reported benefits are sustained (and what type and level of intervention might promote this). However, notwithstanding these limitations, we believe that our research indicates that the potential of written information on fatigue and rheumatic disease is yet to be fully realized, and offers some useful pointers as to how such resources might be used to greater effect.

## 5 CONCLUSION

Fatigue is a common symptom of autoimmune rheumatic diseases, with a significant impact on health‐related quality of life. Patients struggle to understand this symptom and get little support to manage its effects. Written information, in the form of a booklet, can change how patients think about fatigue. This is valuable, alleviating a range of concerns and equipping them to improve their management behaviours. Dissemination of written information by professionals, guided reflection with sensitive challenge, and a clear commitment to follow‐up encourage patients to convert changes in thought to changes in behaviour. For maximum effect, written information needs to be embedded within the conversations and practices of routine outpatient care. Used in such a way, it offers a low‐cost tool for addressing as yet unmet patient needs.
